# Quadricuspid aortic valve repair with a modified‐tricuspidization technique

**DOI:** 10.1111/echo.15448

**Published:** 2022-09-22

**Authors:** Antonio D'Errico Ramirez, Enrico Squiccimarro, Micaela De Palo, Tommaso Acquaviva, Aldo Domenico Milano

**Affiliations:** ^1^ Cardiac Surgery Unit Department of Emergency and Organ Transplantation University of Bari Bari Italy; ^2^ Cardiac Surgery Unit Department of Medical and Surgical Sciences University of Foggia Foggia Italy; ^3^ Cardio‐Thoracic Surgery Department Heart & Vascular Centre Maastricht University Medical Centre Maastricht The Netherlands

**Keywords:** aortic valve regurgitation, aortic valve repair, quadricuspid aortic valve

## Abstract

**Introduction:**

Quadricuspid aortic valve (QAV) is an extremely rare developmental abnormality with an incidence of 0.006%. QAV is an incidental finding that in some patients (23%) may determine aortic regurgitation (AR). Altogether 16% of patients indeed require surgery with AR being the most frequent indication.

**Methods and results:**

We describe a case report of a 46 year‐old female affected by severe aortic regurgitation due to QAV successfully treated with a  modified‐tricuspidization technique associated with cusp extension, prolapsing commissure suturing, and sub‐commissural annuloplasty.

**Discussion:**

QAV repair represents an attractive perspective to overcome the drawbacks of either mechanical or biological prosthesis.

## 1 CASE REPORT

**FIGURE 1:**
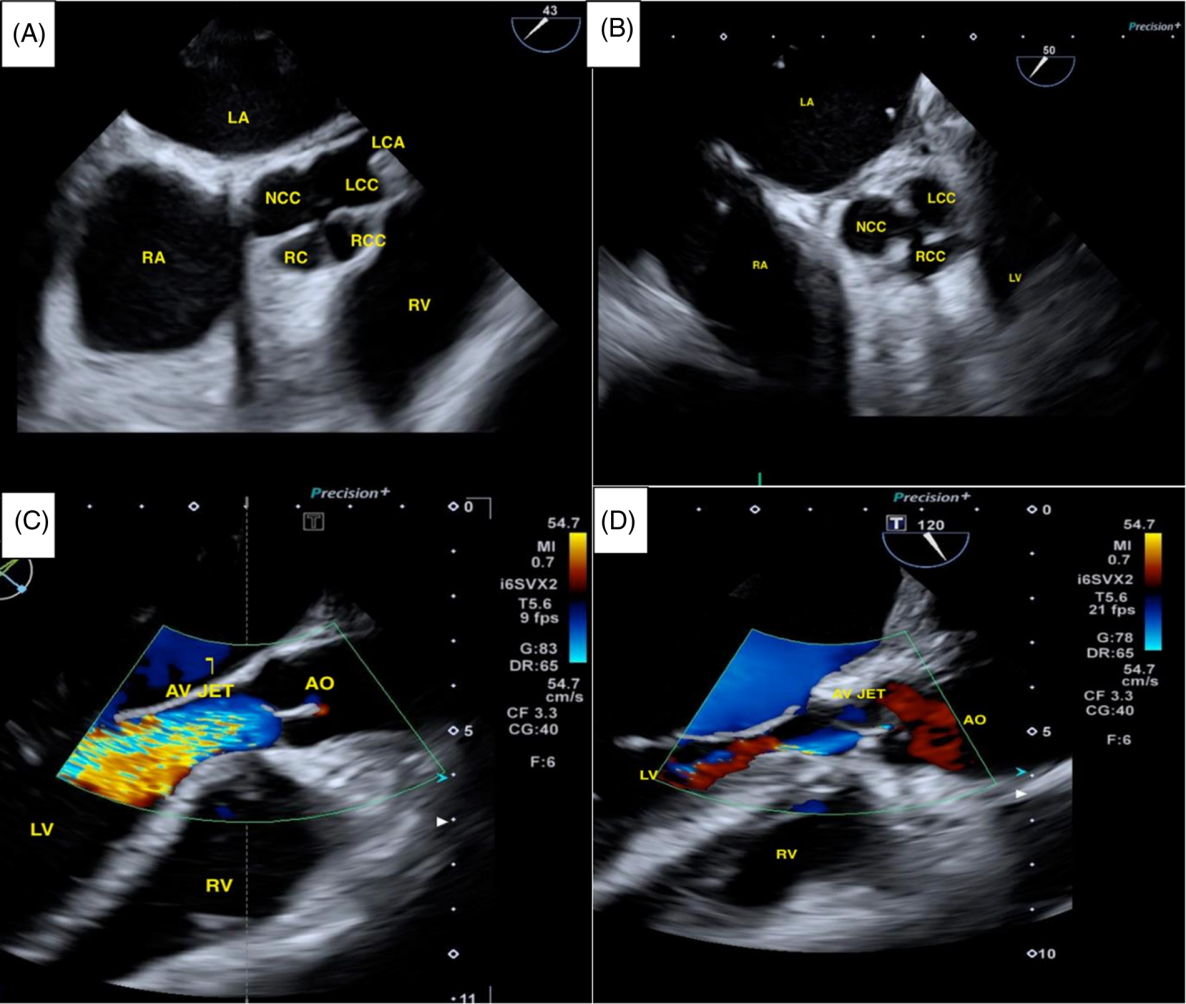
(A) Transesophageal echocardiogram showing Quadricuspid aortic valve with a rudimental cusp (RC) bond with right coronary cusp (RCC). (B) Transesophageal echocardiogram: tricuspidization of aortic valve after repair: left coronary cusp (LCC), non coronary cusp (NCC) and a unique right coronary cusp (RCC). (C) Transesophageal echocardiogram: insufficiency jet of QAV. (D) Mild residual aortic regurgitation after QAV repair. LA, left atrium; LV, left ventricle; RV, right ventricle; LCA, left coronary artery; AO, aorta; AV JET, aortic valve regurgitation doppler‐jet.

**FIGURE 2:**
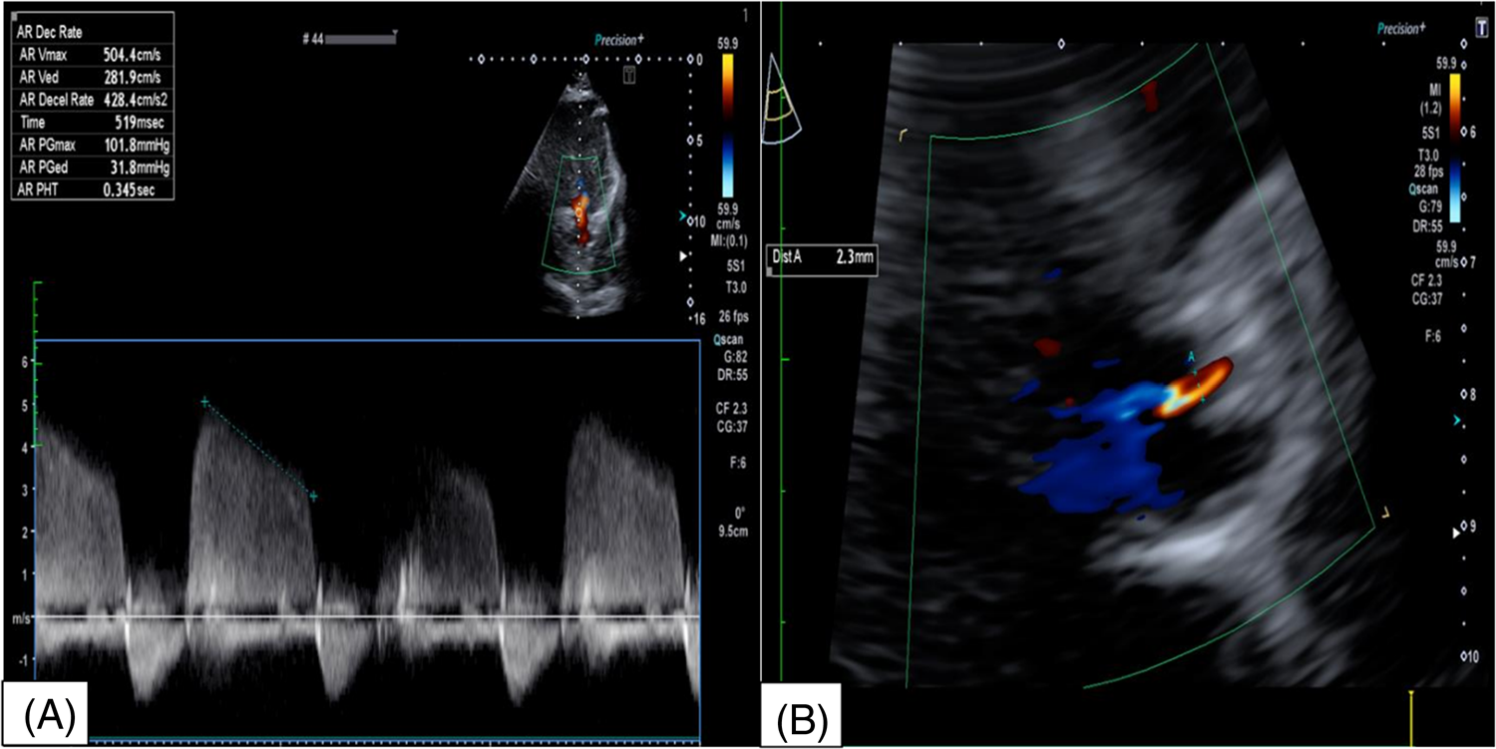
(A) Pre operative. Quantification of QAV regurgitation with pressure half‐time. (B) Follow up transthoracic echocardiogram, quantification of residual aortic valve regurgitation with vena contracta width after QAV repair.

A 46‐year‐old woman was referred to our department for severe and symptomatic aortic regurgitation highlighted on outpatient transthoracic echocardiography (TTE) control. The patient complained of worsening dyspnea over the previous months (New York Heart Association – NYHA Class II). She presented with hypertension and a smoking history. Physical examination revealed a 3/6 diastolic murmur at the 3^rd^ intercostal space on the left sternum border. A transesophageal echocardiogram (TEE) was performed demonstrating as follows: preserved biventricular function and normal heart structures except for a quadricuspid aortic valve determining severe aortic regurgitation with a central jet (vena contracta width – vena contracta width 12 mm, pressure half‐time – pressure half time 345 msec) (Figures 1A,C and 2A), neither the aortic anulus nor the ascending aorta was dilated. After a thorough team analysis and discussion with the patient who refused a mechanical prosthesis due to lifestyle issues, she was scheduled for elective QAV repair.

Following anesthesia induction, a conventional median sternotomy was accomplished. Cardiopulmonary bypass (CPB) was commenced via central aortic and right atrial appendage cannulation, both the aortic root and the left ventricle were vented, aortic cross‐clamp was performed and immediately ensued by the antegrade delivery of cold‐blood cardioplegia into the coronary ostia which was re‐dosed every 20 min.

**FIGURE 3:**
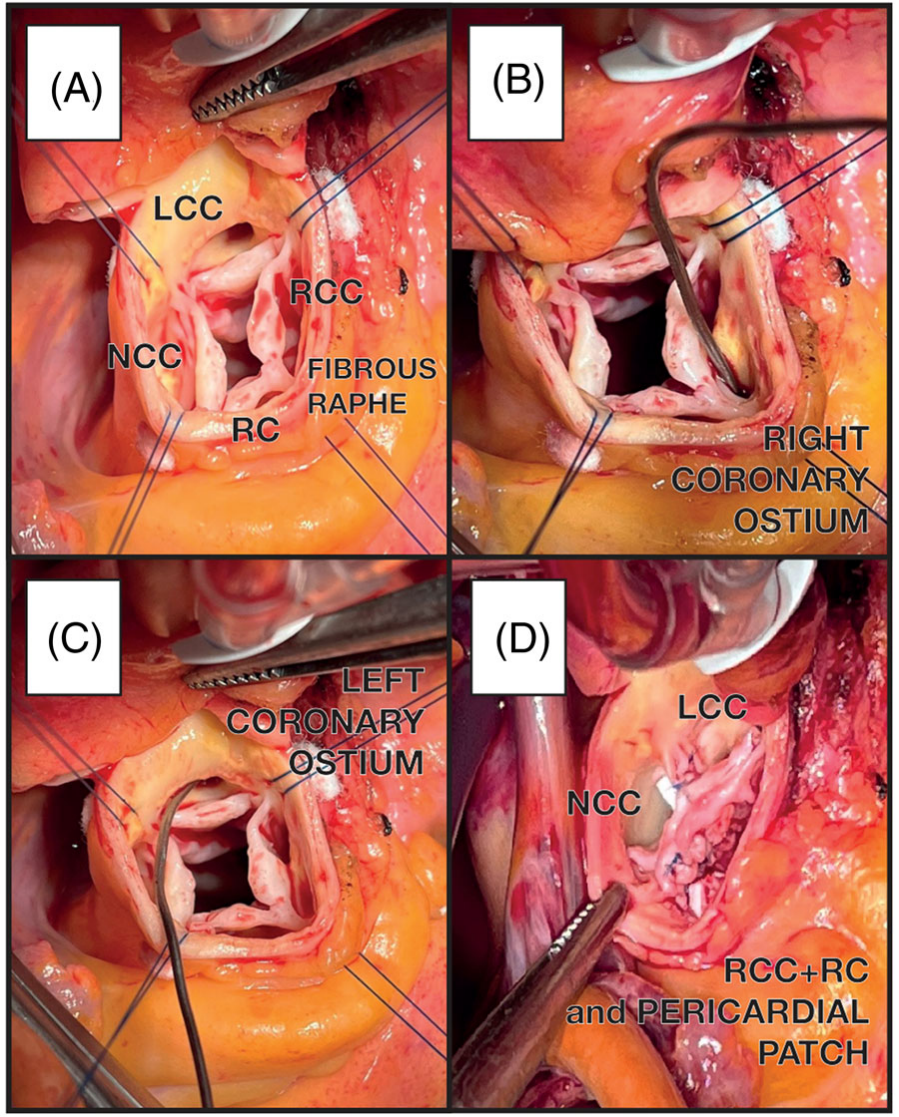
(A) Quadricuspid aortic valve with a rudimental cusp (RC) bond with right coronary cusp with a fibrous raphe (FR) creating a fourth rudimental commissure. (B) Specimen in right coronary ostia. (C) Specimen in left coronary ostia. (D) Final result: good coaptation of the three cusps.

**FIGURE 4:**
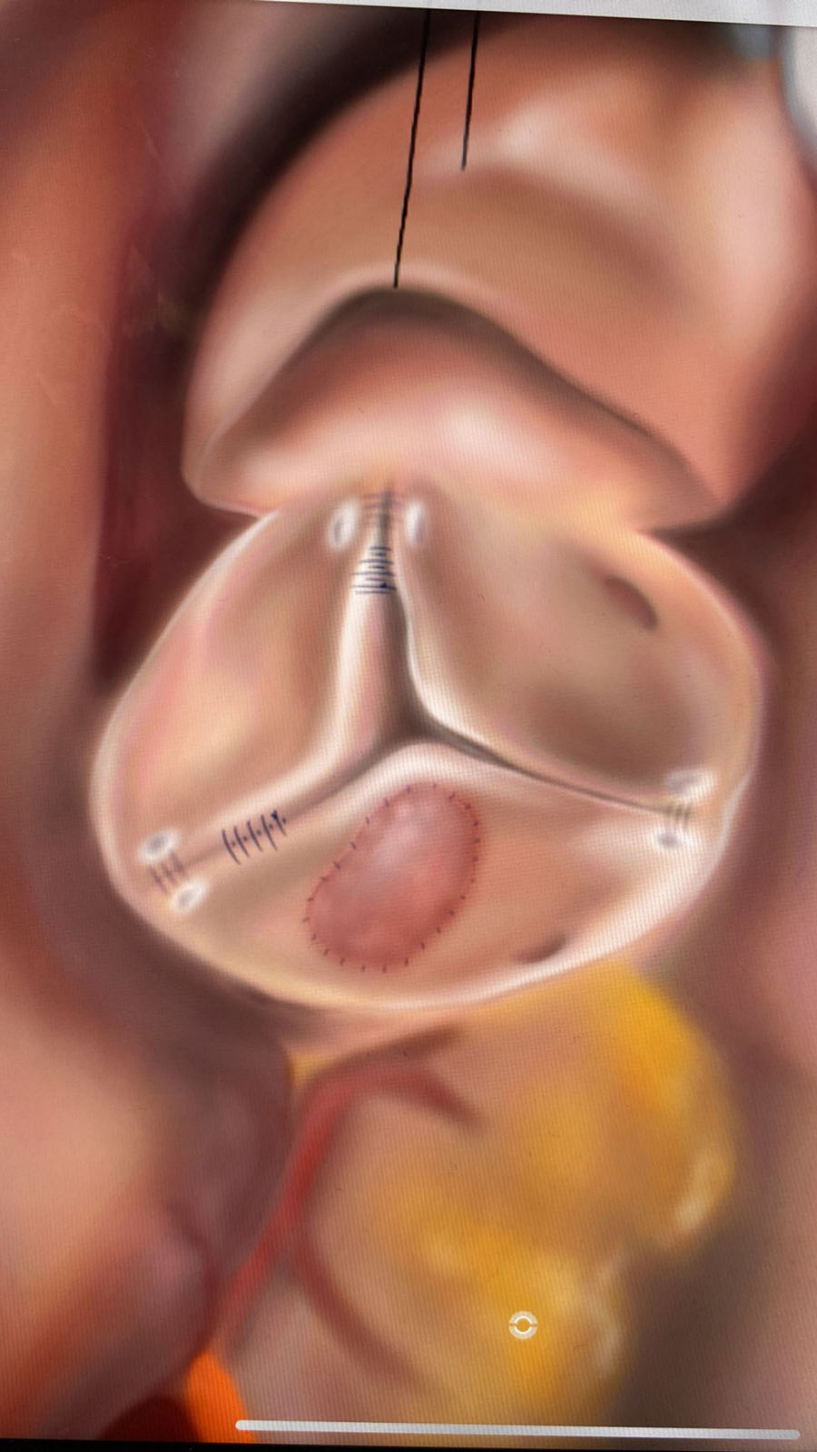
Graphical illustration of the modified‐tricuspidization technique: cusp extension, prolapsing commissure suturing, and sub‐commissural annuloplasty.

A transverse aortotomy 1 cm above the sino‐tubular junction allowed the exposition of the aortic valve apparatus which underwent precise ascertainment for repair's feasibility. QAV diagnosis was confirmed by the surgical finding of a “type‐F” QAV in the Hurwitz and Roberts classification^1^ characterized by two equally‐sized larger cusps (right coronary and non‐coronary – RCC and NCC) and two unequal smaller cusps (left coronary and rudimental – LCC and RC), all appearing thickened especially at the free margin level. A large fenestration was present at RC‐NCC commissure causing the commissure to prolapse. Smaller fenestrations were also seen on the other commissures (Figure 3A–C). Hence, a combined type II (cusp prolapse) and type III (cusp retraction) mechanism was identified as the basis of regurgitation, therefore a modified tricuspidization‐technique entailing raphe‐shaving, prolapsing commissure suturing, cusp repair with a pericardial patch (i.e., cusp extension), and sub‐commissural annuloplasty was performed. First, three commissural traction stitches (4‐0 polypropylene) were placed at the tip of each commissure. Cusps’ free margin thinning was then performed. In order to increase cusp mobility, RC was detached from the aortic wall by shaving a particularly fibrotic raphe (FR). This maneuver revealed a large fenestration of about 1 cm. Consequently, an adequately‐sized pericardial patch was sutured with a 7‐0 polypropylene finally achieving tissue continuity and tricuspidization via the creation of a unique cusp formed by RC and RCC with a “bridging” small pericardial patch. At this point, the prolapsing and fenestrated commissure between RC and NCC was sutured with a running 7‐0 polypropylene as well as the NCC‐LCC commissure, all addressing the issue of cusps’ height of coaptation. Finally, a sub‐commissural annuloplasty of the three commissures with 2‐0 pledged polyester stitches was performed aiming to stabilize the repair (Figures 3D and 4; Video S1).

Conclusive cusps’ re‐assessment for lingering prolapses, coaptation's height/depth of coaptation, and symmetry was thoroughly carried‐out before the aortorrhaphy. Intra operative TEE showed a satisfactory surgical result with a successful QAV repair (coaptation height 7 mm) and a mild residual central regurgitation Figure (1B and D). Ergo, weaning from CPB was obtained without any pharmacologic support and the chest was closed. The postoperative course was uneventful, and the patient was discharged home in good condition on a post‐operative day 7, so is at 3‐month follow‐up: complete recovery from surgery, normal heart function, mild residual central regurgitation, and good quality of life (Figure 3B).

## 2 DISCUSSION

QAV is a rare and usually trivial occurrence but its association with AR, other structural defects, and less commonly with aortic stenosis (only 8% of patients), typically presenting in young patients makes the scenario of absolute clinical relevance.^2^ When surgery is advisable, it is mandatory to provide a result that must be as effective as durable. The rarity of the condition affects the available literature which consists of just a few, hardly‐obtained, and scarcely represented (even over a large timespan) surgical case series.^3^ Overall, a standardized surgical approach to this pathology is lacking and valve replacement represents the most frequent surgical choice (73%) according to the largest surgical case series^3^ of QAV (*n* = 31 patients) even though the shortcomings of either biological or mechanical prosthesis are likely to worsen patients’ quality of life due to valve‐related risks such as thromboembolism, prosthetic valve degeneration or endocarditis, and bleeding. Whereas QAV repair has been demonstrated as feasible and effective in small series (23% of patients undergone surgery for QAV) with medium‐term follow‐up and it is highly appealing given the typical QAV presentation in youth.^3^ In 1981 tricuspidization technique was firstly described with suturing of the rudimentary cusp with the right coronary cusp.^4^ Further various case reports present aortic valve repair with tricuspidization technique. Also, bicuspidization of QAV has been reported^5^ as well as aortic valve reconstruction with autologous pericardium was described.^6^

## 3 CONCLUSION

To the best of our knowledge, we are introducing the first case report of a successful tricuspidization technique associated with a cusp extension technique with a pericardial patch for the treatment of QAV insufficiency, complete pre‐operative intra‐operative and post‐operative imaging is provided.

## CONFLICTS OF INTEREST

The authors declare that there is no conflict of interest.

## Supporting information

Supporting Information
